# Layered Iron Vanadate for High‐Performance and Stable Cathode Material for Aqueous Manganese Batteries

**DOI:** 10.1002/advs.202503006

**Published:** 2025-04-07

**Authors:** Seunghyeop Baek, Dedy Setiawan, Hyeonjun Lee, Sangki Lee, Jangwook Pyun, Seung‐Tae Hong, Munseok S. Chae

**Affiliations:** ^1^ Department of Nanotechnology Engineering Pukyong National University Busan 48547 Republic of Korea; ^2^ Department of Energy Science and Engineering DGIST Daegu 42988 Republic of Korea; ^3^ Research Center for Energy and Environmental Materials National Institute for Materials Science (NIMS) 1‐1 Namiki Tsukuba Ibaraki 305‐0044 Japan; ^4^ Department of Chemistry and Chemical Biology University of New Mexico Albuquerque New Mexico 87131 USA

**Keywords:** aqueous electrolytes, cathode materials, layered iron vanadate, Mn batteries

## Abstract

Aqueous rechargeable metal batteries have gained significant attention because of the low cost, high capacity, and inherent safety offered by nonflammable water‐based electrolytes. Among these, Mn‐based systems are promising owing to their intrinsic stability, abundance, affordability, and high energy density. Despite these advantages, the development of suitable host structures for Mn storage remains underexplored. This study introduces layered iron vanadate, FeV_3_O_9_·1.1H_2_O, as a new cathode material for aqueous Mn batteries, demonstrating exceptional performance. The cathode exhibits a reversible capacity of 306.9 mAh g^−1^ at 0.25 A g^−1^ and an excellent rate performance of 210.6 mAh g^−1^ at 2 A g^−1^. In addition, FeV_3_O_9_·1.1H_2_O exhibits outstanding cycling stability, retaining 73.4% of its initial capacity after 3000 cycles at 3 A g^−^¹, which is attributed to its low layered volume expansion. The underlying reaction mechanism is elucidated through spectroscopic and microscopic analyses. When integrated into the final Mn cell, the cathode system demonstrates superior performance compared to Zn batteries, underscoring its potential for next‐generation aqueous battery systems. These findings advance the aqueous Mn battery technology, paving the way for safer, more cost‐effective, and high‐performance energy storage solutions.

## Graphical abstract

An aqueous Mn battery with a high rate and stable cycle performance is demonstrated using layered iron vanadate as the cathode material. The surface‐capacitive contribution governs the reaction mechanism and exceeds the high‐rate performance of the material. Its small interlayer spacing exceeds that of its stable cycle performance.

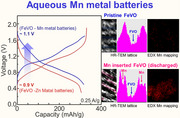

## 1 Introduction

The increasing demand for advanced, environmentally friendly, rechargeable batteries composed of affordable raw materials has led researchers to explore new battery chemistries beyond conventional systems. With global energy storage demands surging toward multi‐terawatt‐hour capacities, lithium‐ion batteries, hindered by limited lithium availability and rising costs, may struggle to meet future large‐scale requirements.^[^ ^1^, ^2^, ^3^ ^]^ These limitations have increased the interest in alternative multivalent‐ion batteries (Ca, Mg, Zn, Al, Mn, and Fe), particularly systems utilizing abundant materials that demonstrate the potential to deliver cost‐effective, safe, and sustainable solutions.^[^ ^4^ ^]^ Among multivalent‐ion batteries, Mn‐ion batteries have gained considerable attention owing to their favorable properties, including high crustal abundance, cost efficiency, and environmental compatibility.^[^ ^5^, ^6^, ^7^, ^8^, ^9^, ^10^ ^]^ Mn‐ion batteries provide opportunities for aqueous electrochemical systems, characterized by enhanced safety, manufacturability, and low internal resistance. Compared with Zn, which has been studied in aqueous batteries worldwide, Mn exhibits a lower redox potential as an anode material while offering superior gravimetric and volumetric capacities (976 mAh g^−1^ and 7250 mAh cm^−3^).^[^ ^6^, ^8^, ^11^, ^12^, ^13^ ^]^

Although aqueous Mn‐ion batteries offer promising features, the range of intercalation‐based electrode materials explored remains limited, with only a few examples including vanadium oxides, Prussian Blue analogs, graphite, and carbonyl‐containing compounds.^[^ ^8^, ^11^, ^14^, ^15^, ^16^, ^17^ ^] ^This finding highlights the urgent need to develop innovative host materials to enhance battery performance. In particular, layered vanadium oxides show considerable potential because of their wide redox range and the flexibility of their layered structures, which can be readily tuned by introducing various intercalants to optimize ion storage and transport.^[^ ^18^, ^19^, ^20^, ^21^ ^]^

Iron vanadates are a naturally occurring group of compounds that exhibit significant structural diversity and are widely found in nature. These materials exist in various hydrated forms, including fervanite (Fe_4_V_4_O_16_·nH_2_O), navajoite (FeV_9_O_24_·nH_2_O), and Kazakhstanite (FeV_3_O_9_·nH_2_O). Among the layered transition metal oxides, kazakhstanite FeV_3_O_9_·nH_2_O has been intensively studied as a cathode material for divalent ion batteries (Ca, Mg, and Zn) and has been reported to exhibit remarkable electrochemical properties.^[^ ^22^, ^23^, ^24^ ^]^ However, its suitability for Mn^2+^ storage remains unexplored.

In this study, we demonstrate FeV_3_O_9_·nH_2_O as a high‐rate and stable host material for aqueous Mn‐ion batteries for the first time. To elucidate the reaction mechanism, we conducted various ex situ structural and elemental investigations using X‐ray diffraction (XRD), energy‐dispersive X‐ray spectroscopy (EDX), and X‐ray photoelectron spectroscopy (XPS). Finally, we compared the Mn and Zn batteries to highlight their potential as next‐generation aqueous batteries.

## 2 Results and Discussion

### 2.1 Synthesis and Material Characterization

**Figure 1:**
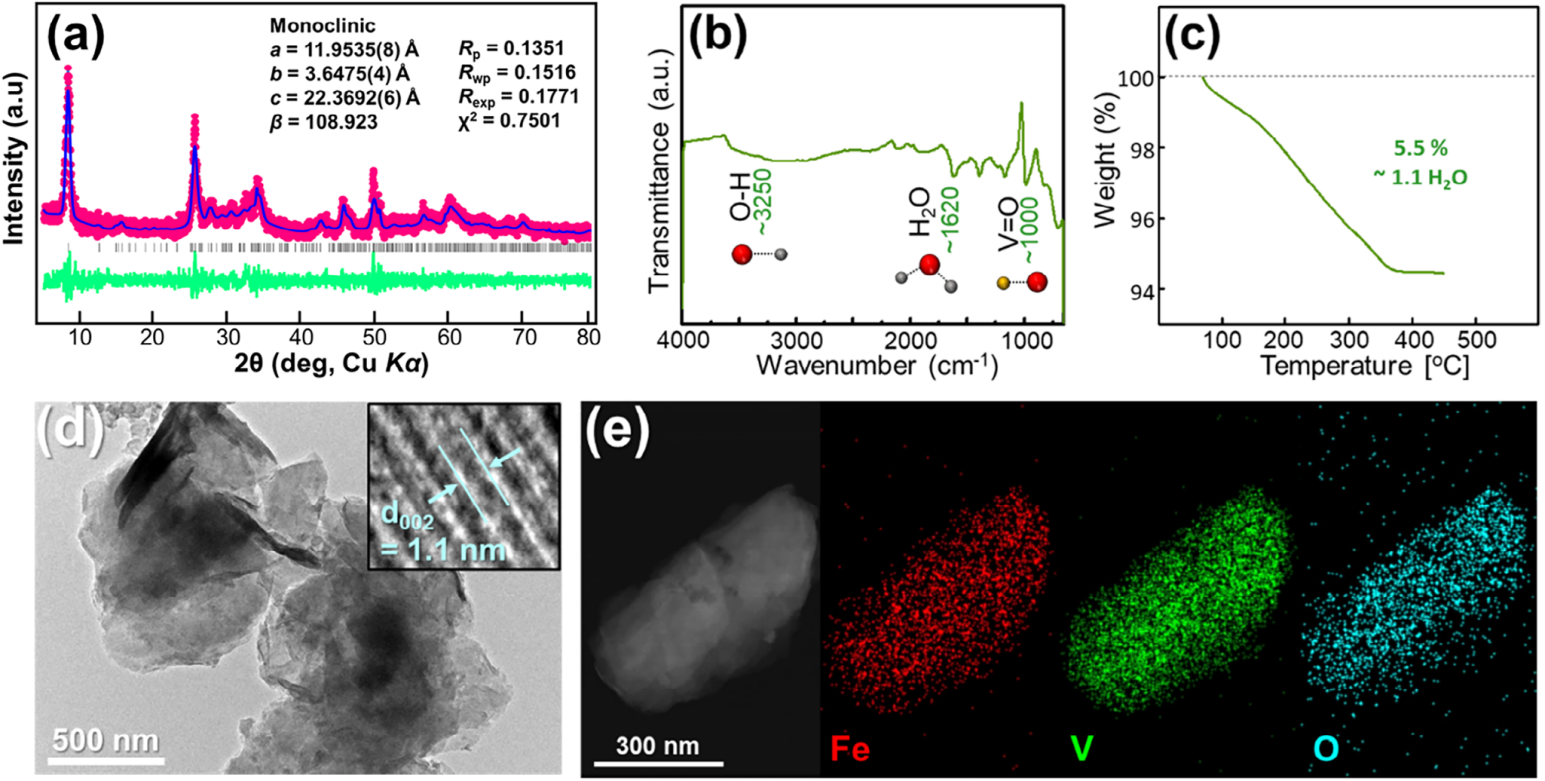
Characterization of FeV_3_O_9_·1.1H_2_O cathode: a) Le Bail fitting of powder X‐ray diffraction (XRD) data, b) Fourier transform infrared (FTIR) data of as‐prepared powder, c) TGA analysis. d) Transmission electron microscopy (TEM) image of FeV_3_O_9_·1.1H_2_O powder and high‐resolution lattice fringe (inset). e) TEM‐energy‐dispersive X‐ray spectroscopy (EDX) elemental mapping for Fe (red), V (green), and O (cyan).

FeV_3_O_9_·nH_2_O was synthesized by the water bath technique, as described in our previous reports.^[^ ^22^ ^]^ The formation of the FeV_3_O_9_·nH_2_O phase was confirmed through Le Bail fitting of the powder XRD data, as shown in **Figure** **1**a. The refined lattice parameters closely matched those of the Kazakhstanite (JCPDS No. 00‐046‐1334). However, its precise structural modeling remains challenging.^[^ ^25^, ^26^ ^]^ The relatively broad peaks of FeV_3_O_9_·nH_2_O indicated its structural determination using powder XRD. However, the single‐crystal growth method for FeV_3_O_9_·nH_2_O remains unknown. The Fe in this structure exists in the +3 oxidation state, while vanadium is in the +5 oxidation state, indicating that further oxidation is not possible. This confirms that Fe is not an intercalated ion but rather an integral part of the structural framework. Furthermore, previous studies have strongly supported this claim by demonstrating that Fe remains within the structure throughout the charge‐discharge process, with no evidence of its removal.^[^ ^24^, ^25^ ^]^

The Fourier transform infrared (FTIR) transmittance spectrum revealed a bond at ≈1000 cm^−^¹ corresponding to the V═O stretching, whereas the absorption bands at 1620 and 3250 cm⁻¹ indicated the presence of adsorbed and crystal water within the host structure, consistent with our previous findings (Figure 1b).^[^ ^22^ ^] ^Several other bands originated from the conductive carbon and PVDF binder in the electrode.^[^ ^24^ ^]^ As shown in Figure 1c, the TGA results indicate that the synthesized FeV_3_O_9_·nH_2_O powder contained 1.1 mol of crystal water. Adsorbed water is usually removed below 200 °C.^[^ ^27^, ^28^ ^]^ High‐resolution transmission electron microscopy (HR‐TEM) images confirmed the nanosheet morphology of the material (Figure 1d and Figure S1, Supporting Information). The TEM image of the nanoflake edges showed layered fringes with a lattice spacing of ≈1.1 nm (Figure 1d, inset). EDX elemental mapping revealed uniform distributions of Fe, V, and O (Figure 1e). To determine the elemental composition, we conducted inductively coupled plasma mass spectrometry analysis, revealing an iron‐to‐vanadium molar ratio of 0.99:3.00, as summarized in Table S1, Supporting Information. Therefore, the as‐prepared compound can be formulated as FeV_3_O_9_·1.1H_2_O.

### 2.2 Electrochemical Mn Storage Performance

**Figure 2:**
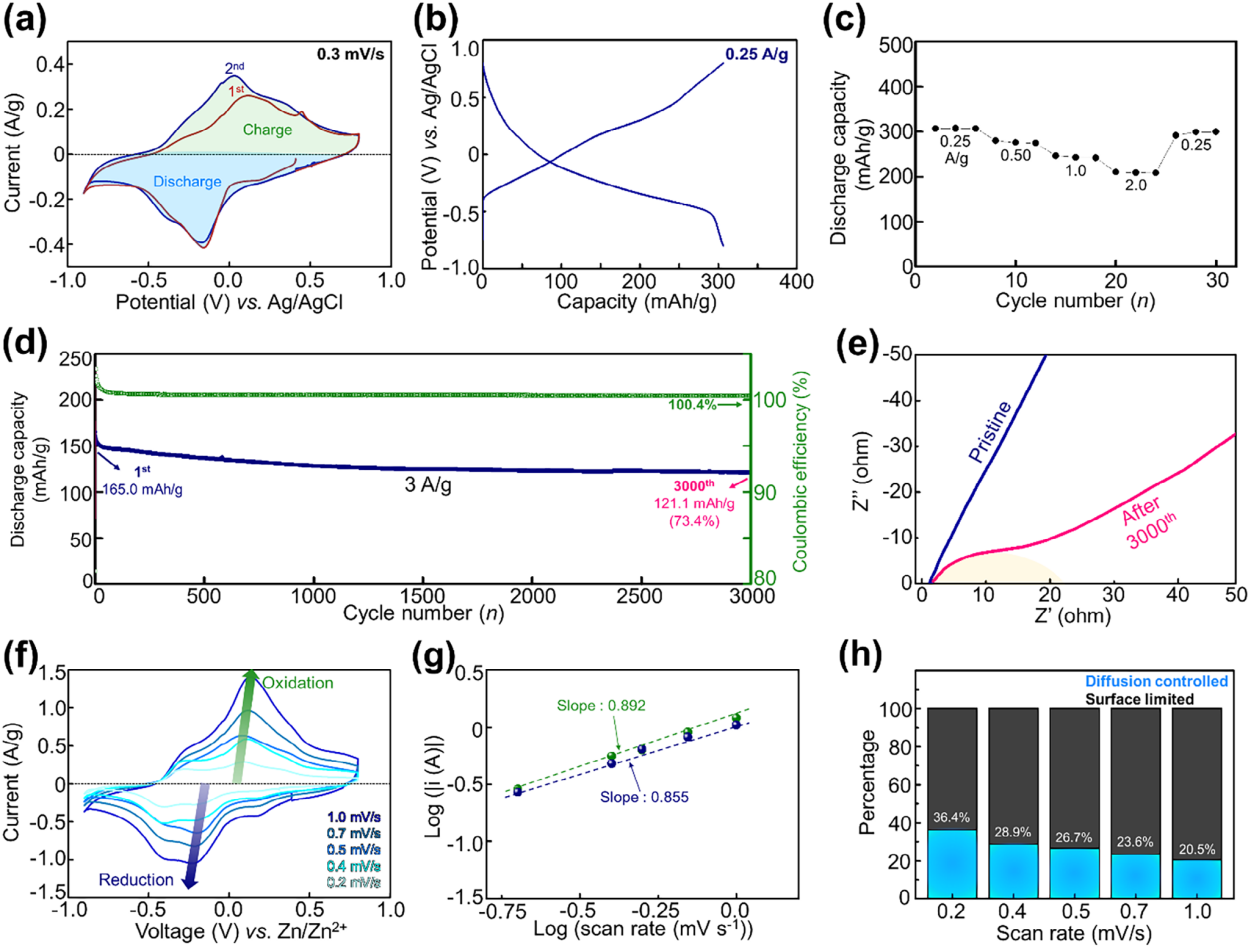
Electrochemical Mn storage performance of FeV_3_O_9_·1.1H_2_O: a) Cyclic voltammetry (CV) curve at a scan rate of 0.3 mV s^−1^. b) Initial discharge–charge profile at 0.25 A g^−1^. c) Rate performance evaluated under varying current densities. d) Long‐term cycling stability tested at 3.0 A g^−1^. e) Impedance spectra measured before and after 3000 cycles. f) CV curves recorded at scan rates ranging from 0.2 to 1.0 mV s^−1^. g) Determination of b values based on the relationship between specific cathodic peak current and scan rate. h) Calculated contributions of diffusion‐controlled (light blue) and surface‐limited (grey) reactions across different scan rates.

The electrochemical Mn ion storage performance of FeV_3_O_9_·1.1H_2_O was investigated using a three‐electrode cell. These cells were constructed using activated carbon as the counter electrode to examine the effects of the anode. When Mn is used, the hydrogen evolution reaction (HER) and the associated side reactions result in increased resistance. **Figure** **2**a shows the cyclic voltammetry (CV) profile of FeV_3_O_9_·1.1H_2_O, exhibiting a distinct and reversible reduction peak at −0.15 V versus Ag/AgCl and an oxidation peak at ≈0.10 V versus Ag/AgCl. In addition, minor reversible peaks were observed, which might be due to proton intercalation, Mn adsorption, or other side reactions originating from electrolytes.^[^ ^14^, ^29^ ^]^ However, it requires more systematic experimental validation to confirm their origin. Notably, the CV profile remained largely unchanged during the second cycle.

The galvanostatic discharge–charge profile of FeV_3_O_9_·1.1H_2_O at 0.25 A g^−1^ is shown in Figure 2b. FeV_3_O_9_·1.1H_2_O exhibited an initial capacity of 306.9 mAh g^−1^ with an average voltage of −0.25 V versus Ag/AgCl. The initial capacity of FeV_3_O_9_·1.1H_2_O suppresses some of the introduced cathode materials, such as Mn_0.18_V_2_O_5_·nH_2_O, Ag_0.33_V_2_O_5_, and organic cathodes.^[^ ^8^, ^11^, ^14^, ^15^ ^]^ The discharge capacities of FeV_3_O_9_·1.1H_2_O at various current densities are shown in Figure 2c. FeV_3_O_9_·1.1H_2_O exhibited remarkable capacities of 280.2, 246.0, and 210.6 mAh g^−1^ at high rate performances of 0.5, 1.0, and 2.0 A g^−1^, respectively (Figure S2, Supporting Information). Figure 2d and Figure S4 (Supporting Information) illustrate the cycling performance of FeV_3_O_9_·1.1H_2_O, demonstrating a capacity of 165.0 mAh g^−1^ at 3 A g^−1^ with 73.4% capacity retention after 3000 cycles. Even after cycling, the crystallinity of FeV_3_O_9_·1.1H_2_O slightly degraded, as shown in Figure S4 (Supporting Information). Figure 2e shows the electrochemical impedance spectroscopy analysis of FeV_3_O_9_·1.1H_2_O in its pristine state after 3000 cycles. After 3000 cycles, the IR resistance increased slightly from 2.4 to 3.2 Ω. The charge‐transfer resistance increased to 22 Ω after prolonged cycling, likely attributed to the formation of a cathode‐electrolyte interphase caused by Mn(OH)_2_ formation from electrolyte decomposition.^[^ ^30^ ^]^

To investigate whether the Mn storage in FeV_3_O_9_·1.1H_2_O fully corresponds to diffusion‐controlled behavior, we conducted CV tests at various scan rates, as shown in Figure 2f. The contributions of the diffusion‐controlled and surface‐capacitive mechanisms were analyzed using the power‐law relationship method.^[^ ^31^ ^]^ The power‐law coefficient, derived from the slope of log(i) versus log(scan rate) in Figure 2g, was ≈0.8 for the oxidation and reduction processes. This intermediate value (between 0.5 and 1.0) suggests that the surface‐capacitive behavior plays a significant role in Mn storage within FeV_3_O_9_·1.1H_2_O. The quantitative analysis in Figure 2h reveals that nearly 64% of Mn storage in FeV_3_O_9_·1.1H_2_O could be attributed to the surface‐capacitive behavior, with the remaining 36% attributed to the diffusion‐controlled mechanism. Furthermore, the dominance of the surface‐capacitive behavior increased with higher scan rates. This phenomenon likely accounts for the remarkable rate of performance observed during the charge–discharge processes. Detailed calculation data are shown in Figure S5 (Supporting Information).

### 2.3 Elemental Analyses During Mn Storage

**Figure 3:**
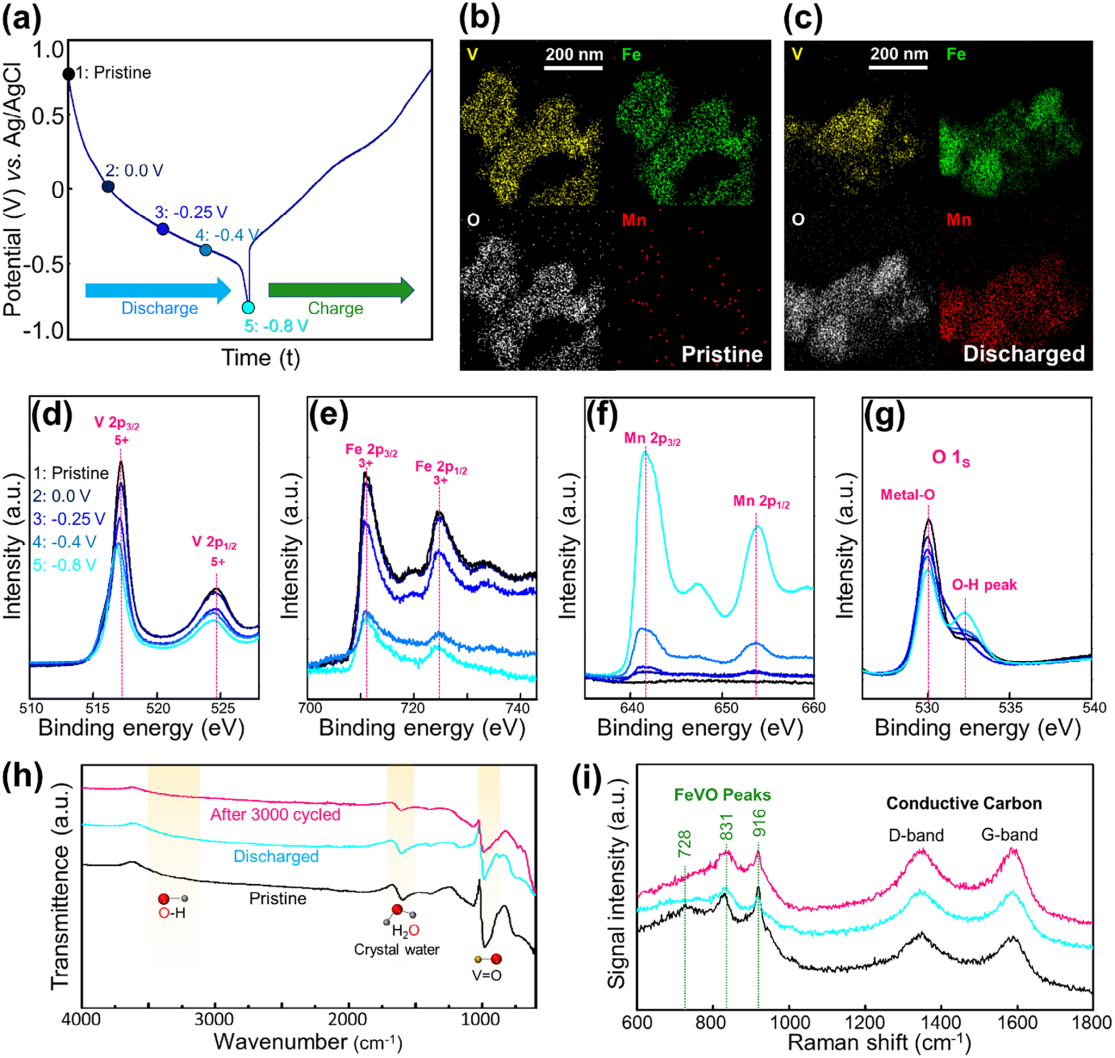
a) Galvanostatic charge–discharge curve from pristine to −0.8 V (vs Ag/AgCl) (measured point), TEM‐EDX mapping of b) pristine and c) fully discharged FeV_3_O_9_·1.1H_2_O. X‐ray photoelectron spectroscopy spectra of d) V 2p e) Fe 3p, f) Mn 2p, and g) O 1s. h) FTIR profile and i) Raman spectra of FeV_3_O_9_·1.1H_2_O at pristine and fully discharged state.

To confirm the Mn storage in FeV_3_O_9_·1.1H_2_O, TEM equipped with EDX and XPS analyses was used, and the results are shown in **Figure** **3**. The discharge–charge profile with the voltage steps is shown in Figure 3a. The TEM‐EDX mappings of Mn in the pristine and fully discharged states are shown in Figure 3b,c, respectively, along with the V, Fe, and O mappings for comparison. Although Mn was initially absent in the pristine state, it was uniformly distributed after full discharge, indicating successful Mn storage in FeV_3_O_9_·1.1H_2_O without phase separation.

The XPS survey spectra of V 2p, shown in Figure 3d,e, reveal the overall reduction of V 2p and Fe 2p during discharge, possibly owing to the formation of electrolyte side products on the surface of the electrode. Figure S6 (Supporting Information) shows the activation of V^5+^/V^4+^ during discharge, with a decreased intensity of V^5+^ 2p_3/2_ peak at 517.2 eV and a new V^4+^ 2p_3/2_ peak emerging at 516.4 eV. Similarly, the Fe^2+^ peak at 709 eV emerged as the intensity of the Fe^3+^ peak at 712 eV decreased. These changes suggested that Mn storage in FeV_3_O_9_·1.1H_2_O involved the reduction of V^5+^ to V^4+^ and Fe^3+^ to Fe^2+^.^[^ ^32^ ^]^ Based on the theoretical capacity of FeV_3_O_9_·1.1H_2_O (71.95 mAh g⁻^1^ per 1‐electron transfer), the first discharge capacity (306.9 mAh g⁻^1^) suggests a 4.26‐electron transfer process. This level of electron transfer would imply the complete reduction of Fe^3+^ to Fe^2+^ and the reduction of V^5+^ to V^4+^, with partial conversion to V^3+^. However, XPS analysis reveals that not all Fe^3+^ was reduced to Fe^2+^, nor was all V^5+^ reduced to V^4+^, indicating that a portion of the discharge capacity is associated with the formation of side products. Quantifying Mn and proton intercalation remains challenging reliably, as elemental analysis of the discharged electrode may not accurately distinguish between Mn incorporated into the structure and surface Mn from the side product.

**Figure 4:**
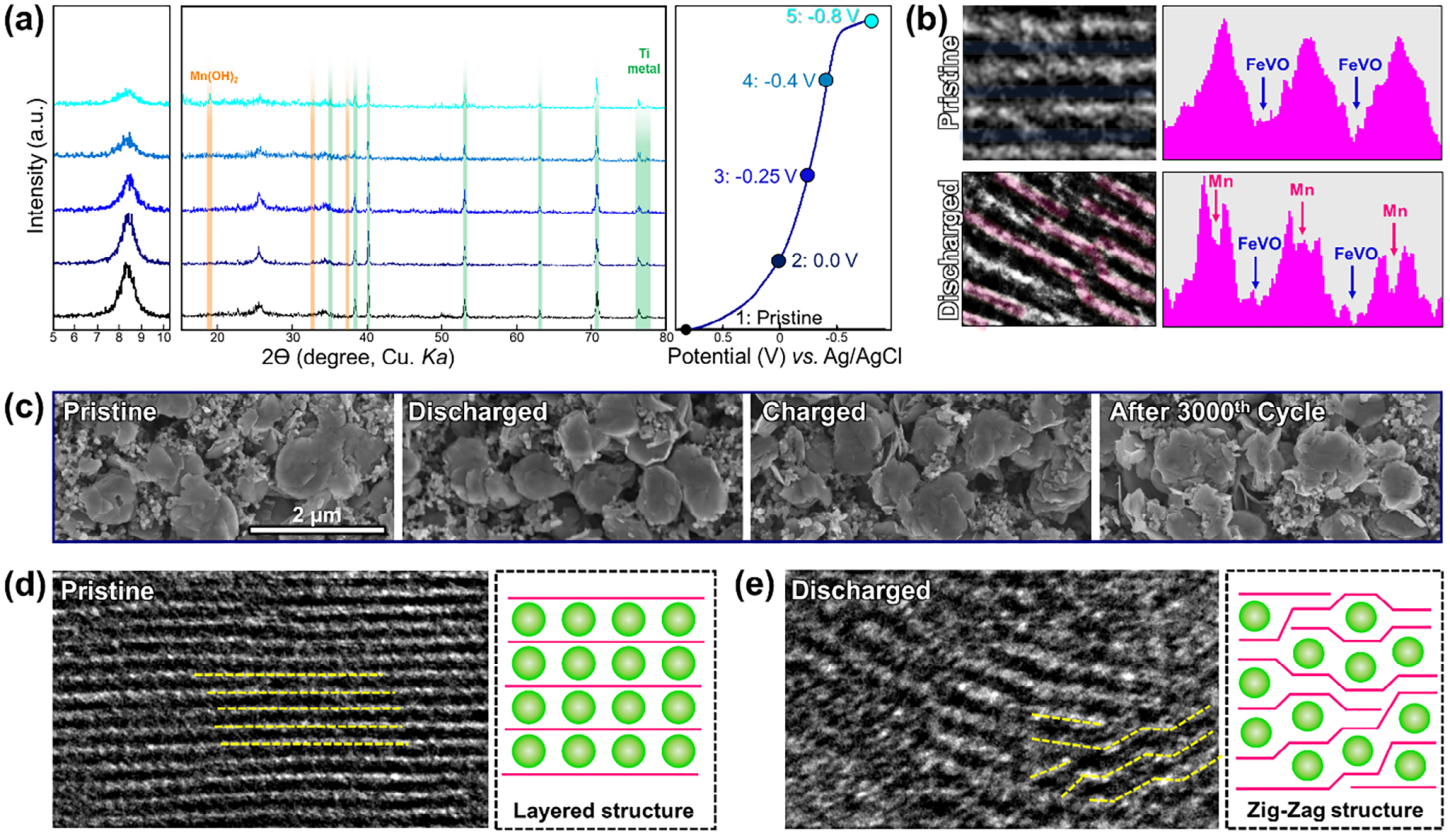
a) Ex situ XRD profile during Mn insertion, along with their discharge–charge profile, b) high‐resolution TEM images for pristine and discharged samples, c) SEM image of FeV_3_O_9_·1.1H_2_O at pristine, 1st discharge, 1st charge, and after 3000 cycles. TEM image of lattice fringe of FeV_3_O_9_·1.1H_2_O at d) pristine and e) after discharge.

Further evidence of Mn storage is provided by the Mn 2p spectra (Figure 3f), which exhibit a significant increase during discharge. Notably, the O 2p spectra show an increase in the O─H peak, potentially corresponding to Mn(OH)₂ as the side product during discharge (Figure 3g). This increase in the O─H species is corroborated by the FTIR spectra recorded after discharge, as shown in Figure 3h. Even after 3000 cycles, the structures remained well‐preserved, with the V═O FTIR peaks exhibiting minimal changes. The V═O peaks shift to a higher wavenumber after the discharge process, indicating that the V═O bonds become stronger during this process. This change in bonding strength may contribute to reduced structural flexibility, leading to the breakdown of the layered structures and the formation of zigzag structures after discharge as shown in **Figure** **4**e. In addition, the Raman peaks confirm that the structural features of FeV_3_O_9_·1.1H_2_O were largely retained, exhibiting only minor degradation (Figure 3i), which is in good agreement with the XRD data shown in Figure S4 (Supporting Information).

Overall, the reaction mechanism in FeV_3_O_9_·1.1H_2_O could be expressed as follows,

(1) $$\begin{array}{ccc} & & \mathrm{Fe}V_{3}O_{9}\cdot 1.1H_{2}O\left(s\right)+\mathrm{xM}n^{2+}\left(\mathrm{aq}\right)+yH^{+}\left(\mathrm{aq}\right)+\left(x+y\right)e^{-}\to \\ & & \;Mn_{x}H_{y}\mathrm{Fe}V_{3}O_{9}\cdot 1.1H_{2}O \end{array}$$

(2) $$Mn^{2+}\left(\mathrm{aq}\right)+2H^{+}\left(\mathrm{aq}\right)+2OH^{-}\left(\mathrm{aq}\right)\to \mathrm{Mn}{\left(\mathrm{OH}\right)}_{2}\left(s\right)+2H^{+}\left(\mathrm{aq}\right)$$

XRD analysis was performed to investigate the structural changes in FeV_3_O_9_·1.1H_2_O during Mn intercalation, as shown in Figure 4a. The intensity of the XRD peaks of FeV_3_O_9_·1.1H_2_O gradually decreased during discharge, likely attributed to the formation of Mn(OH)_2_ as the side product, as corroborated by the XPS survey spectra. Structurally disordered reactions likely occurred. During discharge, the (002) peak at 8.5° shifted progressively to a higher angle (8.7°), indicating a reduction in the FeV_3_O_9_·1.1H_2_O interlayer spacing during Mn^2+^ intercalation. The shrinking of interlayer spacing during Mn^2+^ ion intercalation should be caused by the strong Coulombic attraction of Mn^2+^ ions with the anion framework. A similar phenomenon was also found in the case of Mg^2+^ intercalation into H_2_V_3_O_8._^[^ ^33^ ^] ^This observation is further supported by the TEM images of the layered fringes shown in Figure 4b. After the discharge, a slightly low‐intensity Mn signal was observed between the FeV_3_O_9_·1.1H_2_O layers. Notably, interlayer shrinkage did not affect the overall morphology of the FeV_3_O_9_·1.1H_2_O primary particles. As shown in Figure 4c, no significant changes were observed in the particle structure after discharge, charge, or 3000 cycles. These findings highlighted the remarkable structural stability of FeV_3_O_9_·1.1H_2_O during Mn storage. To understand these structural changes, we performed an HR‐TEM analysis. We observed an interesting peak in the FeV_3_O_9_·1.1H_2_O lattice after discharge (Figure 4d,e). The FeV_3_O_9_·1.1H_2_O lattice evidently showed a disordered lattice fringe upon discharge compared with the pristine state. This finding was in good agreement with the broadened and recurring sharp peaks observed in the XRD patterns during discharge.

**Figure 5:**
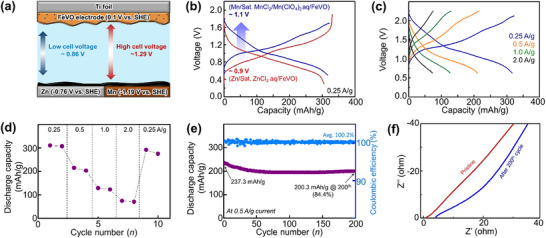
a) Schematic of Zn and Mn batteries with a FeV_3_O_9_·1.1H_2_O cathode, b) galvanostatic charge–discharge profile at 0.25 A g^−1^, c,d) rate performance at different current densities, e) long‐term cycling performance with Mn anode, and f) impedance spectra of pristine cell and after 200 cycles.

Finally, to compare the potential of the Mn‐based system with that of conventional Zn batteries, we conducted tests using an Mn anode. Theoretically, combining a Zn anode with a FeV_3_O_9_·1.1H_2_O cathode results in a discharge voltage of ≈0.86 V, whereas pairing a Mn anode with the same cathode yields an enhanced voltage of 1.29 V, as illustrated in **Figure** **5**a. Tests were performed on each system, and the galvanostatic charge–discharge profiles were obtained, as shown in Figure 5b. Although both systems demonstrated similar capacities of ≈305 mAh g^−1^, the Mn anode notably increased the operating voltage from 0.9 (Zn) to 1.1 V (Mn), confirming its contribution to higher energy density in the aqueous battery system. Since the theoretical redox potential difference of Mn (−1.19 V vs SHE) and Zn (−0.76 V vs SHE) is 0.43 V, while the difference between open circuit voltage and average discharge voltage of both cell is only ≈0.2 V, there must be an influence from each electrode potentials in both systems. Therefore, it is still difficult to conclude whether the voltage discrepancy is simply caused by the Mn and Zn anode or the Mn^2+^ and Zn^2+^ insertion potential into FeV_3_O_9_·1.1H_2_O. Future investigation using a three‐electrode cell is required to systematically unravel this phenomenon. Above all, this result highlights the potential of Mn‐ion secondary batteries as next‐generation energy storage devices.

Figure 5c,d depicts the rate capabilities of these systems. Although the performance was reasonable, the Mn‐based anode system exhibited lower performance than systems utilizing activated carbon anodes. This finding can be attributed to the formation of a resistive layer at the anode interface, which reduces the rate of performance. These findings suggest that further research is required to address the anode interface issues in aqueous systems. However, considering only the FeV_3_O_9_·1.1H_2_O cathode, it demonstrates stable performance at both high and low current densities, as supported by the electrochemical data shown in Figures 2 and 5.

When cycling the Mn battery at 0.5 A g^−1^, the system maintained stability. The initial specific capacity was 237.3 mAh g^−1^, and 84.4% of this capacity was retained after 200 cycles, as shown in Figure 5e. However, the Coulombic efficiency was unstable in the early cycles, likely because of the HER at the anode. Over time, the formation of a stable SEI layer on the anode surface contributed to the system's stabilization.

The impedance measurements before and after cycling are shown in Figure 5f. The IR resistance slightly increased, and the charge‐transfer resistance increased significantly even after the 200th cycle. This finding indicated the presence of a resistive layer on the anode surface. Therefore, to commercialize Mn batteries, it is crucial to stabilize the anode and control the formation of resistive layers through advanced electrolyte engineering. This study demonstrates the considerable potential of Mn batteries as a promising avenue for early‐stage research.

## 3 Conclusions

In this study, FeV_3_O_9_·1.1H_2_O was demonstrated as a new cathode material for aqueous Mn batteries. FeV_3_O_9_·1.1H_2_O exhibited exceptional rate performance, exhibiting 306.9 mAh g^−^¹ at 0.25 A g^−^¹ and 210.6 mAh g^−^¹ at 2 A g^−^¹. This finding also demonstrated excellent cycling stability, retaining 73.4% of its capacity after 3000 cycles at 3 A g^−1^. Through structural and spectroscopic characterizations, we observed highly stable atomic and microstructural behaviors of FeV_3_O_9_·1.1H_2_O during Mn storage. Elemental analyses confirmed Mn storage in FeV_3_O_9_·1.1H_2_O, accompanied by the formation of Mn(OH)_2_ species, primarily attributed to proton intercalation.^[^ ^14^, ^34^ ^]^ Mn intercalation was also observed in the HR‐TEM images, suggesting that Mn^2+^ ions were simultaneously inserted into the FeV_3_O_9_·1.1H_2_O structure. Structural analyses revealed a reduction in the interlayer spacing and disordered phase after discharge. However, even after 3000 cycles, the structures were well maintained with minor degradation.

In addition, we extensively compare the Mn batteries and Zn‐based systems, highlighting the potential of Mn batteries as next‐generation aqueous energy storage solutions. Mn batteries exhibited higher operating voltages than the Zn batteries. However, further advancements are required to stabilize Mn in aqueous electrolytes for practical applications.

Our study significantly contributes to the understanding of cathode materials for Mn‐ion batteries. Notably, this is the first known report that explores FeV_3_O_9_·1.1H_2_O as a cathode material for Mn‐ion batteries, a subject that has been rarely addressed in previous research. These insights have the potential to drive innovation and advancement in next‐generation cathode materials, paving the way for high‐energy and durable battery systems in the future.

## 4 Experimental Section

### Material Synthesis and Characterization of FeV_3_O_9_·1.1H_2_O

A solution of NH_4_VO_3_ (3 mmol) (99%, Alfa Aesar) was dissolved in 100 mL of distilled water and stirred at 90 °C for 1 h. Subsequently, 10 mL of a 0.1 m Fe(NO_3_)_3_·9H_2_O (98%, Sigma–Aldrich) solution was gradually added to the NH_4_VO_3_ solution, and the resulting mixture was maintained at 90 °C for 24 h. The initially transparent yellow solution became opaque brown owing to the formation of insoluble colloidal products. The brown precipitate was collected by vacuum filtration, washed three times with distilled water, and dried overnight at 80 °C in air.

### Electrochemical Characterization

The cathode was fabricated using a mixture of FeV_3_O_9_·1.1H_2_O powder, Super C conductive carbon (Timcal), and a poly(vinylidene fluoride) binder (Kureha Co.) in an 8:1:1 weight ratio. The components were dispersed in N‐methyl‐2‐pyrrolidone and uniformly coated onto a 32 µm titanium foil (Alfa Aesar). The coated electrodes were dried at 70 °C and pressed using an electrode‐pressing device. For cathode characterization, a three‐electrode configuration was used in a beaker‐type cell. The Ti current collector and a saturated MnCl_2_ aqueous solution served as the electrolyte, whereas an Ag/AgCl electrode was used as the reference. Activated carbon, separated by Whatman glass fiber separator paper, was used as the counter electrode. A two‐electrode configuration was adopted for the Mn‐ and Zn‐ FeV_3_O_9_·1.1H_2_O cell. FeV_3_O_9_·1.1H_2_O cathodes were paired with Mn or Zn anodes, and the electrolyte comprised a 9:1 (v/v) mixture of saturated MnCl_2_ and Mn(ClO_4_)_2_ solutions. While Zn‐ FeV_3_O_9_·1.1H_2_O cell was fabricated using saturated ZnCl_2_ aqueous solutions as an electrolyte. Electrochemical characterizations including CV, galvanostatic charge–discharge testing, and impedance spectroscopy were performed using a Biologic VMP‐3e system. Impedance measurements were performed in PEIS mode over a frequency range of 0.1 Hz to 200 kHz.

## Conflict of Interest

The authors declare no conflict of interest.

## Supporting information



Supporting Information

## Data Availability

The data that support the findings of this study are available from the corresponding author upon reasonable request.
