# Supplementary material for: Layered Iron Vanadate for High‐Performance and Stable Cathode Material for Aqueous Manganese Batteries
Source: Adv Sci (Weinh). 2025 Apr 7;12(26):2503006. doi: 10.1002/advs.202503006 (PMC12245107; doi:10.1002/advs.202503006)
Supplement: Supplementary file 1 — Supporting Information [file ADVS-12-2503006-s001.docx]

Supporting Information

Layered Iron Vanadate for High-Performance and Stable Cathode Material for Aqueous Manganese Batteries

Seunghyeop Baek^+^, Dedy Setiawan^+^, Hyeonjun Lee, Sangki Lee, Jangwook Pyun, Seung-Tae Hong, Munseok S. Chae*


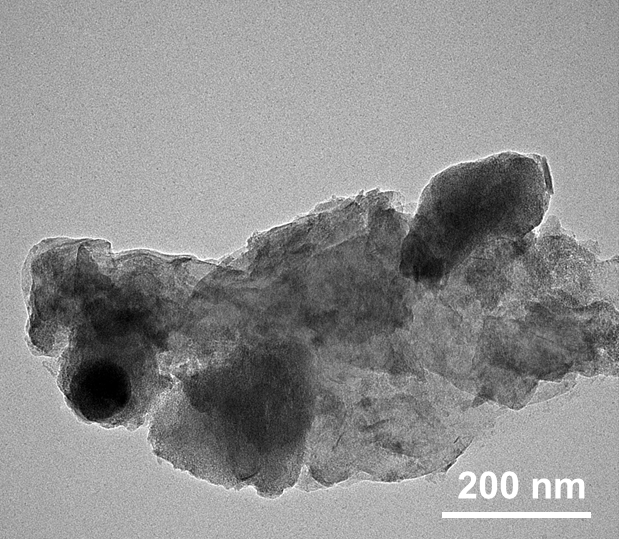


**Figure S1.** Transmission electron microscopy images of FeV_3_O_9_·1.1H_2_O particle.

**Table 1.** Elemental ratios estimated from inductively coupled plasma mass spectrometry analysis for FeV_3_O_9_∙1.1(H_2_O) sample.

|  | Mass ratio (%) | | **Relative atomic ratio** | |
| --- | --- | --- | --- | --- |
|  | Fe | V | **Fe** | **V** |
| Sample |  |  |  |  |
| Synthesized  FeV_3_O_9_·1.1H_2_O | 24.82 | 75.18 | **0.99** | **3.00** |


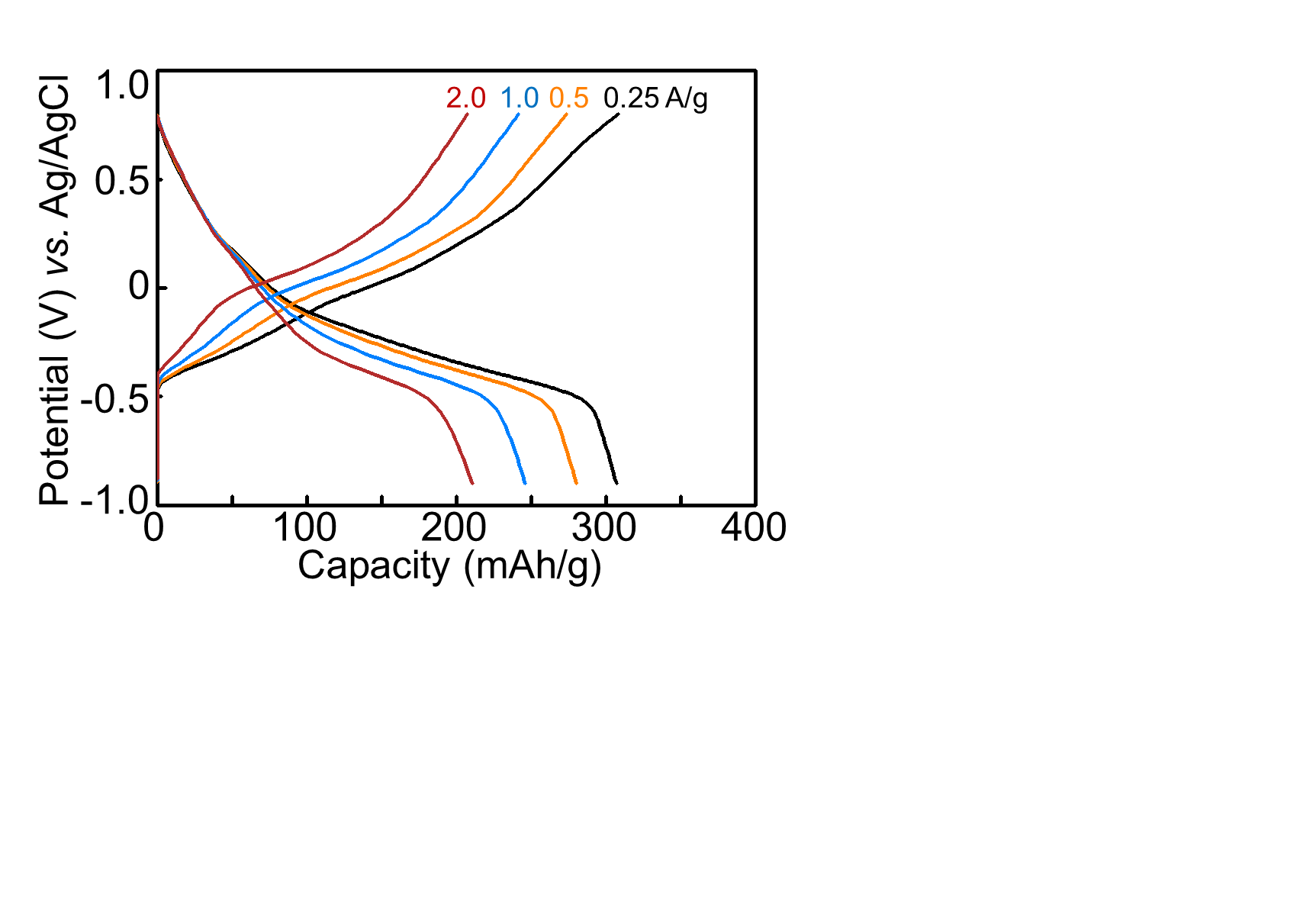


**Figure S2.** Galvanostatic charge–discharge curve at various current densities.


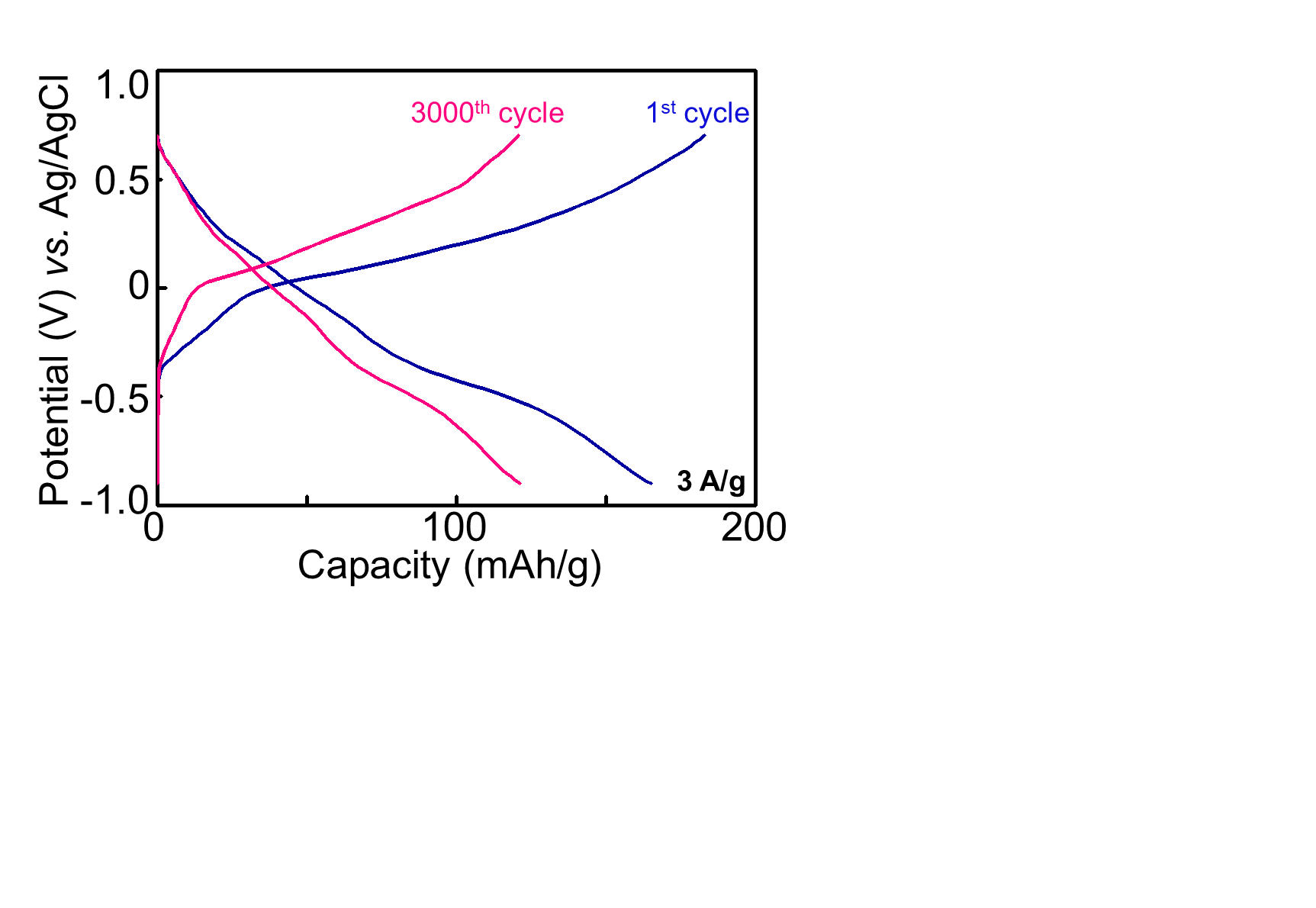


**Figure S3.** Galvanostatic discharge–charge profiles of 1^st^ and 3000^th^ cycles.


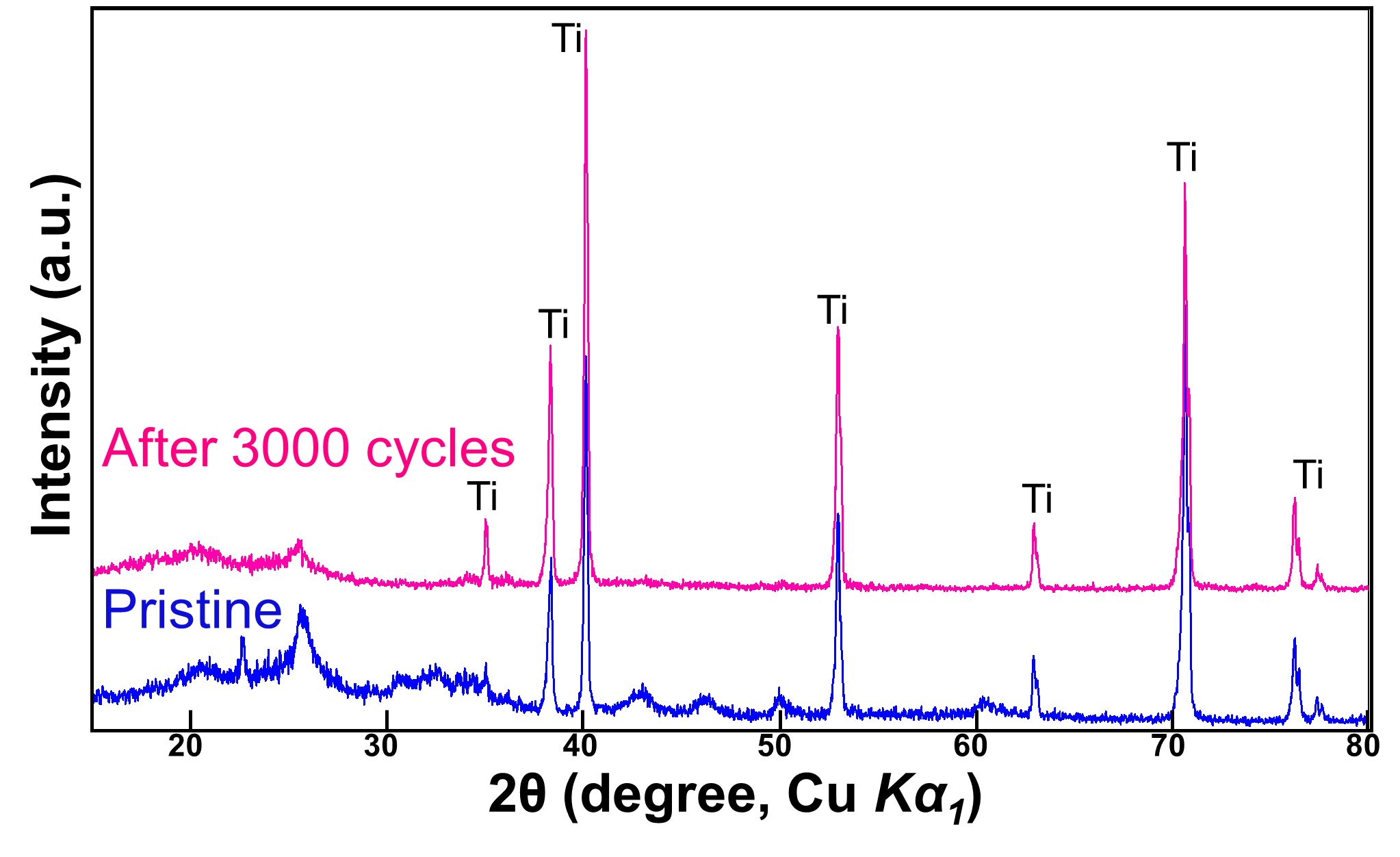


**Figure S4.** X-ray diffraction patterns of pristine electrode and after 3000 cycled electrodes.


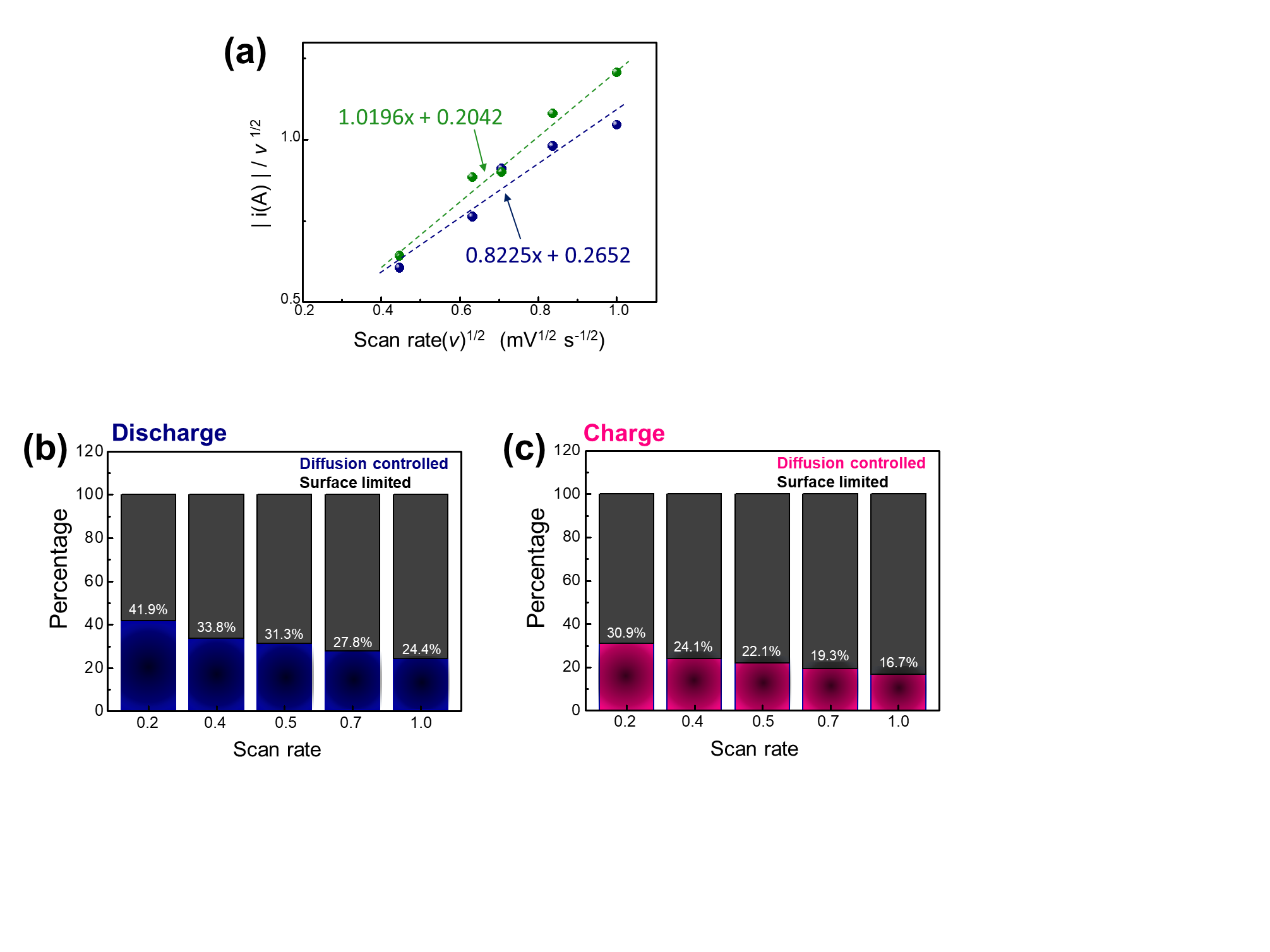


**Figure S5.** (a) Cathodic peak current dependence on scan rate (obtained to determine capacitive and intercalation contributions to energy storage). Calculated intercalation/adsorption ratios with various scan rates for (b) discharge and (c) charge processes.

**
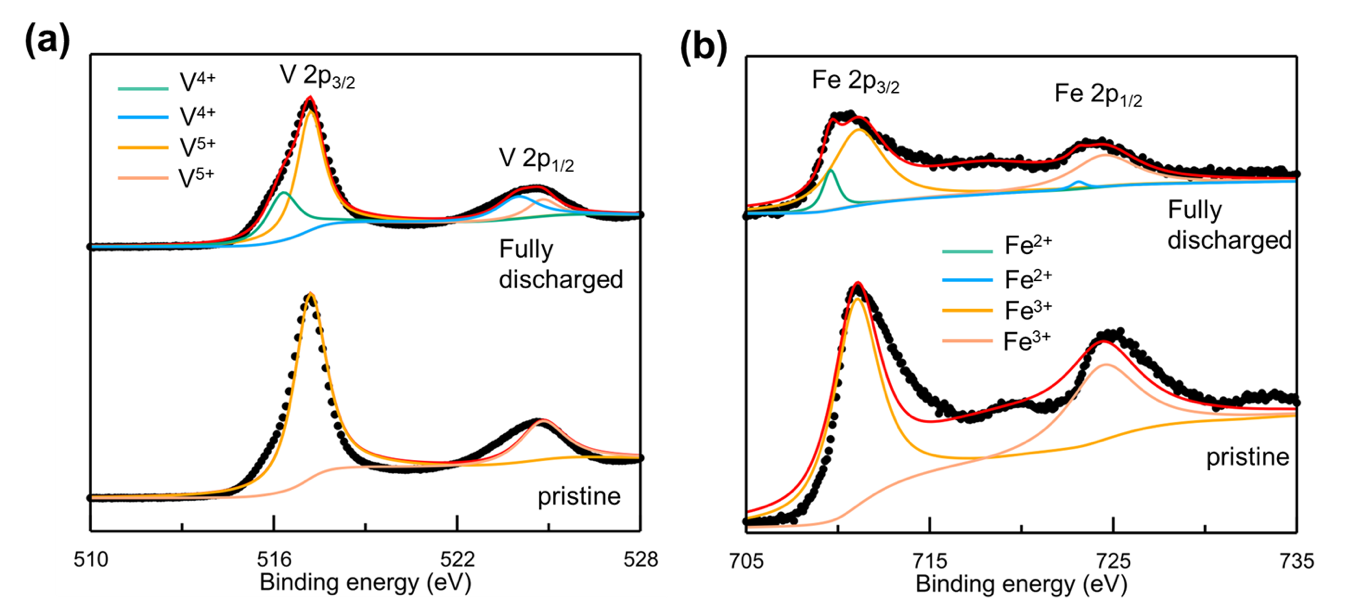
**

**Figure S6.** X-ray photoelectron spectroscopy spectra of pristine and fully discharged FeV_3_O_9_·1.1H_2_O electrodes (a) vanadium and (b) iron.
